# A Novel Family of Winged-Helix Single-Stranded DNA-Binding Proteins from Archaea

**DOI:** 10.3390/ijms23073455

**Published:** 2022-03-22

**Authors:** Can Huang, Xuehui Liu, Yuanyuan Chen, Junshi Zhou, Wenqian Li, Niannian Ding, Li Huang, Jingyu Chen, Zhenfeng Zhang

**Affiliations:** 1 MOE Key Laboratory of Precision Nutrition and Food Quality, College of Food Science and Nutritional Engineering, China Agricultural University, Beijing 100083, China; canhuang_cau@hotmail.com (C.H.); muxiaoqingqing@163.com (W.L.); 2 State Key Laboratory of Microbial Resources, Institute of Microbiology, Chinese Academy of Sciences, No. 1 West Beichen Road, Chaoyang District, Beijing 100101, China; 15539028523@163.com (J.Z.); dingniannian19@mails.ucas.ac.cn (N.D.); huangl@im.ac.cn (L.H.); 3 The Research Platform for Protein Sciences, Institute of Biophysics, Chinese Academy of Sciences, 15 Datun Road, Chaoyang District, Beijing 100101, China; xhliu@ibp.ac.cn (X.L.); chenyy@ibp.ac.cn (Y.C.); 4 College of Life Science, University of Chinese Academy of Sciences, Beijing 100049, China

**Keywords:** winged-helix protein, single-stranded DNA-binding protein, hyperthermophilic archaea, NMR

## Abstract

The winged helix superfamily comprises a large number of structurally related nucleic acid-binding proteins. While these proteins are often shown to bind dsDNA, few are known to bind ssDNA. Here, we report the identification and characterization of Sul7s, a novel winged-helix single-stranded DNA binding protein family highly conserved in *Sulfolobaceae*. Sul7s from *Sulfolobus islandicus* binds ssDNA with an affinity approximately 15-fold higher than that for dsDNA in vitro. It prefers binding oligo(dT)_30_ over oligo(dC)_30_ or a dG-rich 30-nt oligonucleotide, and barely binds oligo(dA)_30_. Further, binding by Sul7s inhibits DNA strand annealing, but shows little effect on the melting temperature of DNA duplexes. The solution structure of Sul7s determined by NMR shows a winged helix-turn-helix fold, consisting of three α-helices, three β-strands, and two short wings. It interacts with ssDNA via a large positively charged binding surface, presumably resulting in ssDNA deformation. Our results shed significant light on not only non-OB fold single-stranded DNA binding proteins in Archaea, but also the divergence of the winged-helix proteins in both function and structure during evolution.

## 1. Introduction

Genomic DNA exists primarily as a duplex, while single-stranded DNA regions are generated transiently by the unwinding of duplex DNA during DNA replication, repair, recombination, and telomere maintenance (1,2). To maintain and protect DNA in the single-stranded (ss) state, single-stranded DNA-binding proteins (SSBs) have been evolved in all known cellular organisms [1,2,3] and some viruses [4,5,6]. These proteins bind ssDNA with high affinity and low sequence specificity, playing essential roles in DNA transactions, primarily by sequestering and protecting transiently formed ssDNA [7,8] and recruiting enzymes to ssDNA [9]. In eukaryotes, SSBs also serve to trigger checkpoint response to DNA damage [10,11].

A major portion of SSBs contains one or more conserved oligonucleotide/oligosaccharide-binding (OB) fold domains, which are responsible for high-affinity sequence-independent ssDNA binding [2,3]. These OB fold containing SSBs, so-called canonical SSBs, presumably share a common ancestor despite their differences in sequence and subunit organization [12,13]. SSBs from most bacteria, such as *Escherichia coli*, function as a homotetramer with each subunit containing a single OB fold [14]. Eukaryotes contain a heterotrimeric SSB complex, termed replication protein A (RPA), which is composed of RPA70, RPA32, and RPA14 subunits. The complex harbors six OB folds, of which two mediate subunit interactions and the remaining four are involved in ssDNA binding [15,16]. The domain architecture of canonical SSBs in Archaea appears to be more complex. Like eukaryotic RPA, euryarchaeal SSB monomers consist of multiple OB folds, forming either heterotrimers or heterodimers [17]. One of the OB folds in each monomer is characteristically interrupted by a zinc-binding domain that is also found in the RPA70 subunit of eukaryotic RPA [18]. On the other hand, crenarchaeal SSBs contain a single OB fold followed by an acidic flexible C-terminal tail that is not involved in ssDNA binding, such as *Sulfolobus solfataricus* SSB (SsoSSB) [19]. However, it should be noted that the crystal structure of the OB fold of SsoSSB bears similarity in structure to the ssDNA-binding domains of human RPA70 and hSSB1, suggesting close phylogenetic relationship between the archaeal and the eukaryotic proteins [20,21].

SSBs without an OB fold domain were also found in some hyperthermophilic archaea. For instance, most species of the Thermoproteales appear to lack the gene encoding an SSB protein with OB folds, and encode a novel ssDNA-binding protein with low sequence specificity instead, termed ThermoDBP, which is structurally distinct from the canonical SSB [22]. The crystal structure of ThermoDBP from *Thermoproteus tenax* reveals a homodimer linked by a coiled-coil leucine zipper. Each monomer contains a unique ssDNA-binding domain with an extended cleft lined with hydrophobic phenylalanine residues and flanked by basic amino acid residues [23]. The replacement of the canonical SSB by ThermoDBP in Thermoproteales suggests that non-canonical SSBs in hyperthermophilic crenarchaea may serve physiological roles identical to those of the canonical SSBs. Intriguingly, although SSB was shown to be essential for cell viability of *S. islandicus* M.16.4 as revealed by the Tn5 transposon mutagenesis analysis [24], it was recently reported that a mutant strain of *S. acidocaldarius* with the sole *ssb* gene deleted exhibited vigorous growth under laboratory conditions [25]. These contradictory results point to the possible presence of non-canonical SSBs functionally complementary (or at least partially complementary) to the canonical SSB in some archaeal species.

In the present study, we report the identification and characterization of a novel small and basic ssDNA-binding protein from *S. islandicus*, denoted Sul7s, which is highly conserved in *Sulfolobaceaea*. Sul7s binds ssDNA with sequence preference for T stretches. It inhibits DNA annealing and causes no changes in the melting temperature of DNA duplexes in vitro. The protein adopts a winged helix-turn-helix fold, contacting DNA through a unique large ssDNA-binding surface, of which the two wings and strand β2 serve critical roles. Our results not only reveal a novel family of ssDNA-binding proteins in hyperthermophilic archaea, but also expand the knowledge of the potential physiological roles of the winged-helix proteins.

## 2. Results

### 2.1. Sul7s Exists as a Thermostable Monomer in Solution

**Figure 1:**
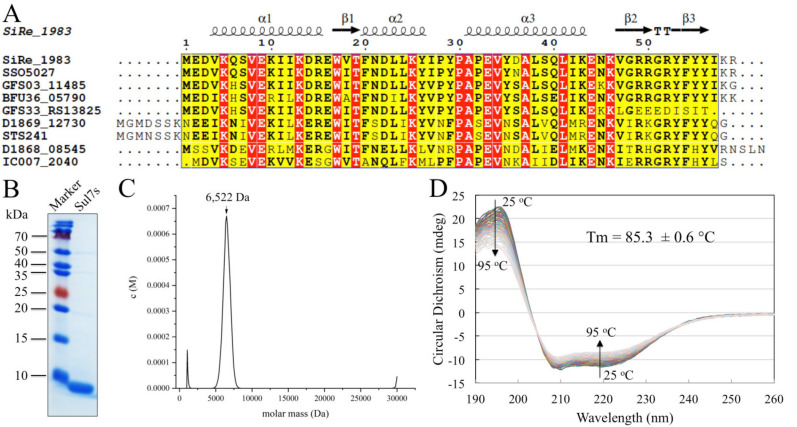
Characterization of the recombinant Sul7s protein. (**A**) Sequence alignment of Sul7s family proteins. Sequences are from *S. islandicus* (SiRe_1983), *Saccharolobus solfataricus* (SSO5027), *Sulfolobus* sp. E5-1-F (GFS03_11485), *Sulfolobus* sp. A20 (BFU36_05790), *Stygiolobus azoricus* (D1868_08545), *Sulfuracidifex tepidarius* (IC007_2040), *Sulfurisphaera ohwakuensis* (D1869_12730), *Sulfurisphaera tokodaii* (STS241) and Sulfolobus sp. E11-6 (GFS33_RS13825). (**B**) SDS-PAGE analysis of the recombinant Sul7s protein purified from *E. coli*. (**C**) Analytical ultracentrifugation of the recombinant Sul7s protein. The calculated apparent molecular mass of Sul7s is indicated. (**D**) CD spectra of Sul7s at temperatures ranging from 25–95 °C.

SiRe_1983 from *S. islandicus* Rey15A contains 59 amino acid residues (theoretical Mr: 7214 Da) and is highly basic (calculated pI: 9.26). The protein has 21 charged residues including seven lysines and five arginines. The protein is predicted to contain a winged helix Storkhead-box1 domain (pfam 10264), which is likely to function in DNA binding [26], suggesting its potential functional role as a DNA-binding protein. Sequence comparison shows that the homologues of SiRe_1983 are found in all genera of the hyperthermophilic crenarchaeon *Sulfolobaceae*, a branch in the Crenarchaeota, except for *Acidianus* and *Metallosphaera*, with sequence similarities above 70% (Figure 1A). The name of this novel protein family was then designated Sul7s, by following the nomenclatures used for the other seven kDa DNA-binding proteins from *Sulfolobus* since it was later shown to be an ssDNA-binding protein.

Recombinant Sul7s without any tag was overproduced in *E. coli* BL21 (DE3) and purified by cation-exchange chromatography following heat-treatment. Gel-filtration chromatography showed a single sharp peak of Sul7s indicating its purity and homogeneity in solution (Figure S1). Further SDS-PAGE analysis of the recombinant protein showed a single band of an expected molecular mass of ~7 kDa with the purity >98% (Figure 1B). The apparent molecular mass of Sul7s was ~6522 Da, as determined by analytical ultracentrifugation, which is slightly smaller than expected, indicating its existence as a monomer in solution (Figure 1C). Far-UV CD spectra of Sul7s, measured at 25~95 °C, revealed the presence of both α and β secondary structure elements (Figure 1D). The melting temperature (Tm) of Sul7s, as calculated based on the changes of the spectra, was 85.3 ± 0.6 °C, indicating that the protein is hyperthermostable in solution.

### 2.2. Sul7s Is an ssDNA-Binding Protein

**Figure 2:**
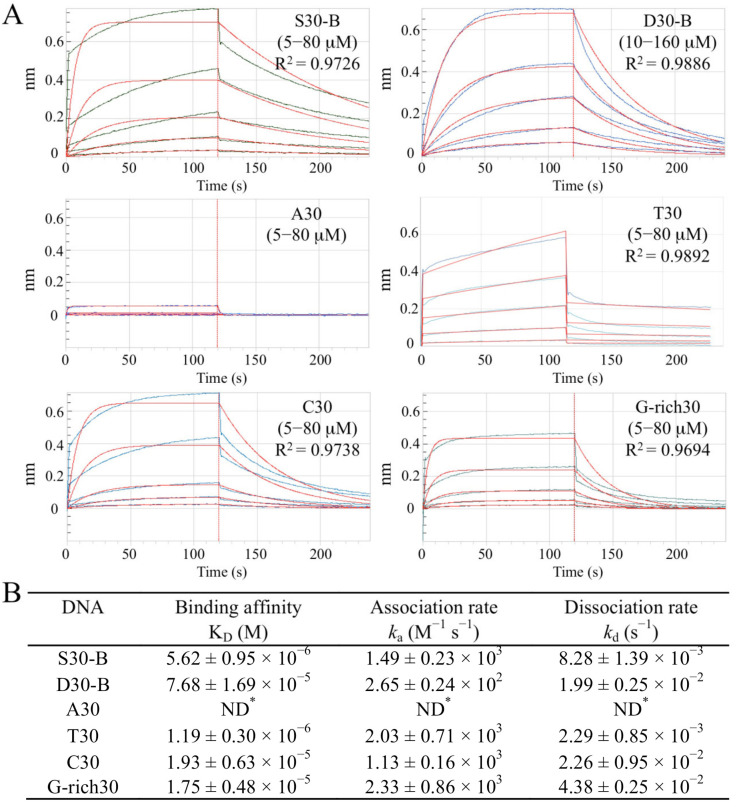
ss- and dsDNA binding properties of Sul7s. (**A**) BLI assays of Sul7s binding to the 30-nt oligonucleotides (S30, A30, T30, C30, and G-rich30) and the 30-bp DNA duplex D30 with the same sequence to S30. The R^2^ value for each fit is indicated. (**B**) Kinetic parameters of the Sul7s binding ss- and dsDNA determined from three independent BLI experiments. ND, not detected.

To investigate DNA binding by Sul7s, gel shift experiments were carried out. Sul7s was capable of generating band shifts on supercoiled, linear, and open circular plasmid pBR322 DNAs (Figure S2A), and showed no apparent topological preference in DNA binding. A similar gel shift pattern was observed for the protein on M13 ssDNA (Figure S2B). Therefore, Sul7s binds both ds- and ssDNA in vitro. We then compared the affinities of Sul7s for dsDNA and ssDNA by using biolayer interferometry (BLI) assays. We found that the apparent dissociation constants (*K_D_*) of Sul7s for a 30-nt oligonucleotide of random sequence (S30) and a dsDNA fragment (D30), prepared by annealing S30 to its complementary strand, were 5.62 ± 0.95 μM and 76.8 ± 16.9 μM, respectively, indicating that the protein preferentially binds to ssDNA over dsDNA in vitro (Figure 2A and Figure S3). Kinetic analysis revealed that Sul7s had a ~six-fold higher association rate for ssDNA than for dsDNA and a ~two-fold lower dissociation rate for ssDNA than for dsDNA, suggesting that the protein was significantly more efficient in ssDNA recognition than in dsDNA recognition, and the Sul7s-ssDNA complex was slightly more stable than the Sul7s-dsDNA complex (Figure 2B).

We then examined the effect of sequence on ssDNA binding by Sul7s (Figure 2A and Figure S3). Among the tested oligonucleotides, A30 was barely bound by the protein under the experimental conditions (Figure 2A). C30 and G-rich30 were bound by the Sul7s with similar affinities (*K_D_* values of 19.3 ± 6.3 μM and 17.5 ± 4.8 μM, respectively), which were ~3.5-fold lower than that for S30 (Figure 2B). By comparison, Sul7s bound tightly to T30 with an affinity ~4.7-fold higher than that for S30 (*K_D_* = 1.19 ± 0.30 μM). It appears that Sul7s preferentially binds T-rich sequences over A-rich sequences in vitro.

**Figure 3:**
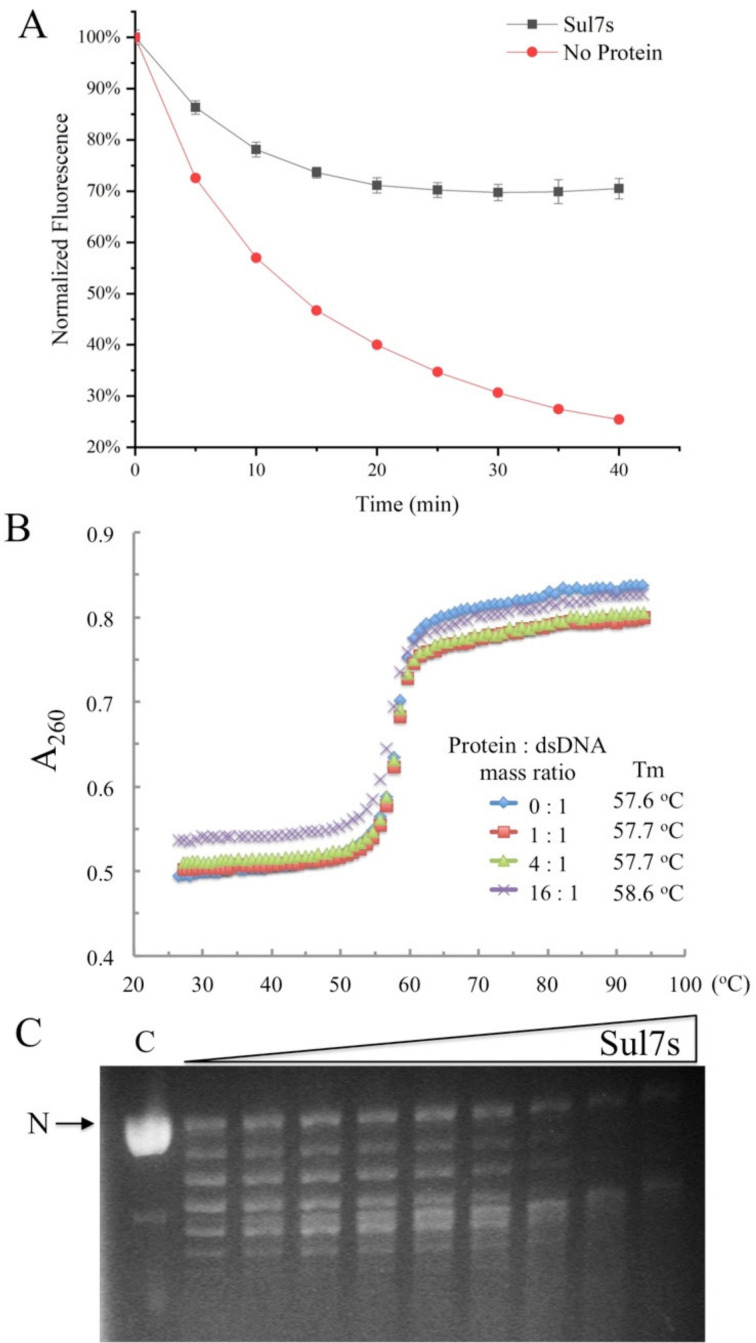
Effects of Sul7s on DNA strands annealing, melting temperature, and DNA supercoils. (**A**) Effects of Sul7s on the kinetics of DNA strands annealing. The 5′-FAM labeled S32-F was mixed with the 3′-BHQ1 labeled S32-R in the absence of Sul7s or presence of Sul7s at the protein:ssDNA molar ratio of 100:1 at 30 °C. The annealing of the DNA strands was measured by monitoring the changes in fluorescence intensity at intervals using a fluorescence spectrophotometer. Changes in normalized fluorescence intensity for each sample were calculated and plotted against time. (**B**) Effect of binding by Sul7s on thermal stability of dsDNA. Thermal denaturation of the 26-bp dsDNA fragment AT26 in the presence and absence of Sul7s was determined by monitoring changes in UV absorbance at 260 nm in a temperature range from 25 to 95 °C on a DU800 UV/Visible spectrophotometer. (**C**) Nick closure analysis of the ability of Sul7s to constrain DNA supercoils. Single-nicked plasmid pBR322 was incubated with Sul7s at protein/DNA mass ratios of 0, 1, 2, 4, 8, 16, 32, 64, 128, and 256, respectively and ligated with T4 DNA ligase at 25 °C. Samples were deproteinized and subjected to agarose gel electrophoresis. N, nicked circular plasmid.

The preferential binding of Sul7s to ssDNA prompted us to look into the effects of the protein on DNA annealing and DNA topology. A pair of complementary DNA strands S32-F and S32-R, labeled with 6-FAM and BHQ1 at the 5′- and 3′-end, respectively, were mixed and incubated with Sul7s, and the fluorescence was monitored over time (Figure 3A). In the absence of Sul7s, the annealing reaction was nearly completed within 40 min, evidenced by a ~75% decrease in normalized fluorescence intensity. By contrast, less than 30% decrease in fluorescence was observed in the presence of Sul7s at the protein:ssDNA molar ratio of ~100:1, where the DNA is supposed to be saturated by the protein, suggesting that Sul7s efficiently inhibited DNA strand annealing. Intriguingly, however, the melting temperature (Tm) of a 24-bp AT-alternating DNA duplex remained largely unchanged following the addition of Sul7s (Figure 3B), indicating that Sul7s did not influence the thermal denaturation of dsDNA. On the other hand, nick-closure assays showed that Sul7s was unable to constrain DNA supercoils (Figure 3C). From these results, we infer that Sul7s do not influence the stability or conformation of dsDNA, but interferes with annealing between complementary DNA strands.

### 2.3. Solution Structure of Sul7s Reveals a Winged-Helix Fold

The solution structure of Sul7s was determined based on 1388 NOE derived distance restraints, 52 phi and 51 psi dihedral angle restraints derived from multidimensional NMR spectroscopy (Table 1). The ^1^H-^15^N HSQC spectrum of the Sul7s showed excellent signal dispersion and uniform signal intensities (Figure S4), indicating the existence of a predominant conformation. This ensured the signal assigning process with the completeness of resonance assignment of 96.9, 79.8, and 67.4% for elements hydrogen, carbon, and nitrogen, respectively.

**Figure 4:**
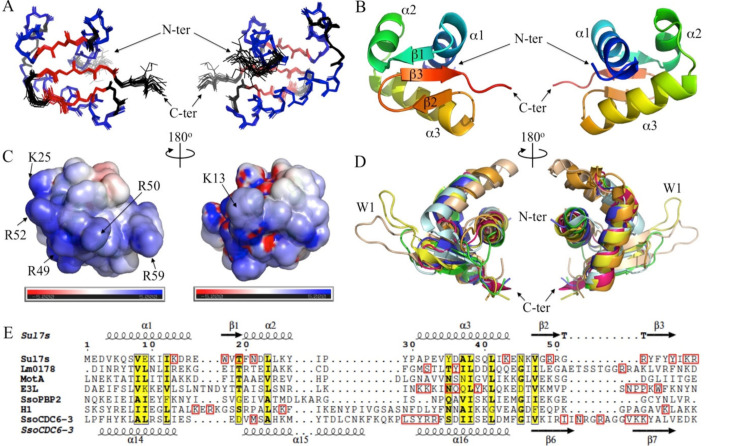
Overall solution structure of *S. islandicus* Sul7s. (**A**) Ribbon representation of the 20 lowest-energy solution structures of Sul7s. The backbone of α-helices, β-strands, and random coils are shown in blue, red, and black, respectively. (**B**) Cartoon representation of Sul7s. (**C**) Molecular surface of Sul7s colored by electrostatic potential. Blue, positively charged; red, negatively charged; white, neutral. Some positively charged residues on the molecular surface are labeled. (**D**) Superposition of Sul7s (in blue) with Lmo0178 from *L. monocytogenes* (in yellow, PDB code: 5F7P), bacteriophage T4 DNA-directed RNA polymerase subunit MotA (in pink, PDB code: 6K4Y) [34], the Z-DNA-binding domain of E3L from Yatapoxvirus (in green, PDB code: 1SFU), PBP2 from *S. solfataricus* (in pale cyan, PDB code: 5N35), the globular domain I of yeast Histone H1 (in orange, PDB code: 1UST) and CDC6-3/Orc1-3 from *S. solfataricus* (in wheat, PDB code: 2QBY). (**E**) Structure based sequence alignment of the proteins shown in (**D**). The residues contacting DNA for each protein are indicated by red boxes.

The overall structure of Sul7s is well defined (Figure 4). Sul7s folds into a compact globular structure composed of a right-handed three-helix bundle (helix α1: V4-R15, α2: F20-Y26 and α3: A31-E44) and a three-strand antiparallel β-sheet (strand β1: W17-T19, β2: V47-R50 and β3: Y53-Y56) in the order of α1-β1-α2-α3-β2-β3 (Figure 4A,B). The side-chains of most of the hydrophobic residues (V4, I11, I12, L23, L24, V34, L38, L41, I42, and V47) of Sul7s are involved in the interactions between the secondary structure elements, ensuring tight packing within the structure, which may contribute greatly to the hyperthermostability of the protein. The N-terminal region (M1-D3) and the C-terminal region (K58-R59) of the protein are unstructured and highly flexible (Figure 4A). Side chains of K13, R15, K43, R49, R52, K58, and R59 face outward, forming a positively charged region on the electrostatic surface of the protein (Figure 4C). Most of these residues are highly conserved (Figure 1A), indicating that proteins of the Sul7s family have similar structures as well as surface charge distributions.

Topologically, the structure of Sul7s is quite similar to those in the winged helix DNA binding domain superfamily (InterPro entry: IPR036390, www.ebi.ac.uk/interpro) [30]. A typical winged helix domain is a compact α/β structure consisting of two large wings (W1 and W2), three α-helices (α1, α2, and α3) and three β-strands (β1, β2, and β3), arranged in order α1-β1-α2-α3-β2-W1-β3-W2 (PMID: 10679470) [31]. However, Sul7s lacks the two large wings and possesses a small hinge (G51-R52) between β2 and β3 and a short flexible C-terminal loop (I57-R59) at the corresponding positions (Figure 4D). A search for structural homologs using DALI server [32] yielded numerous hits of winged helix DNA binding domain containing proteins with diverse biological functions related to DNA metabolism. Transcriptional regulators predominate among these proteins. For example, Lmo0178 from *Listeria monocytogenes* (PDB code: 5F7P) [33], the most relevant hit, is a repressor, open reading frame, kinase (ROK) transcription regulator that may function to mobilize transcription of cycloalternan (CA) pathway. It binds to the intergenic regions upstream of the *lmo0178* and *lmo2446* start codons as a dimer, and the binding is stabilized by the inducer α-1,6-linked disaccharide isomaltose (an intermediate in CA catabolism). Nearly all residues in Sul7s are structurally aligned to those of Lm0178 by Dali with the secondary elements of each other fitting very well (Figure 4E). The RMSD between Sul7s and Lm0178 aligned by Dali is 1.0 Å (with a Z-score of 10.1), indicating a high structural similarity between the two proteins, although they share a very low identity at the amino-acid sequence level (lower than 20%). Besides transcription factors, representative structural homologs of Sul7s in the top relevant hits include bacteriophage T4 DNA-directed RNA polymerase subunit MotA (with a Z-score of 9.2) (PDB code: 6K4Y) [34], the Z-DNA-binding protein domain of E3L from Yatapoxvirus (with a Z-score of 9.0) (PDB code: 1SFU) [35], a small subunit (PBP2) associated with the replicative DNA polymerase I from *Saccharolobus solfataricus* (with a Z-score of 8.3) (PDB code: 5N35) [36], the globular domain I of yeast Histone H1 (with a Z-score of 6.9) (PDB code: 1UST) [37], and the cell division control protein 6 homolog 3 (CDC6-3/Orc1-3) from *S. solfataricus* (with a Z-score of 6.5) (PDB code: 2QBY) [38], with RMSD of 1.6, 1.7, 1.8, 1.8, and 2.0 Å, respectively (Figure 4D). Again, Sul7s shows a low similarity at the amino-acid sequence level with these proteins (generally lower than 25%) despite the high structural homology (Figure 4E). Among the structural homologs of Sul7s mentioned above, most have been reported to contribute to DNA binding either by themselves or in a complex, but none of them were shown to interact with ssDNA directly. Together, Sul7s may represent a novel member of the winged helix DNA binding domain superfamily that is distantly related to other protein families at both levels of the amino-acid sequence and the biological functions.

### 2.4. Sul7s Possesses a Unique ssDNA-Binding Surface

**Figure 5:**
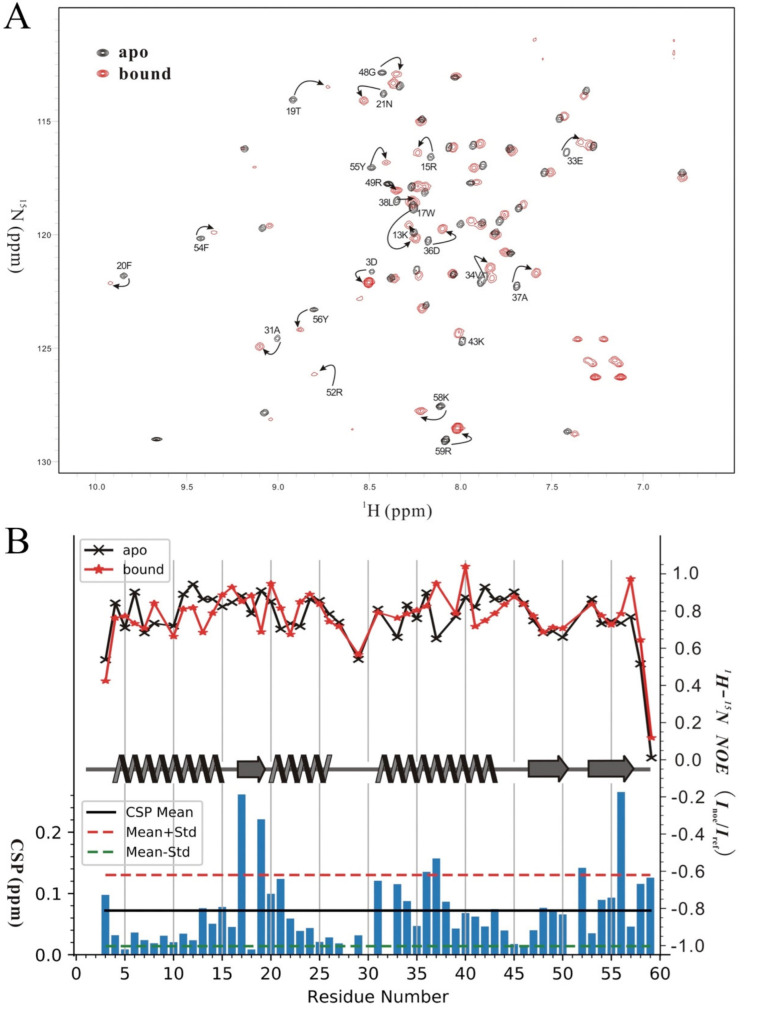
NMR CSP analysis of Sul7s upon ssDNA binding. (**A**) Overlaid ^1^H-^15^N HSQC spectra of apo (in black) and bound (in red) states of Sul7s. (**B**) Chemical shift perturbations (lower part) and ^1^H-^15^N hetero-nuclear NOE (upper part) versus residue numbers. The mean value of all residues’ CSP is drawn in black solid line, and CSP+Std and CSP-Std values are drawn in red and blue dashed lines. In the hetero-nuclear NOE plot, the symbols and lines for the apo and bound form of Sul7s are drawn in black and red colors, respectively. The secondary structures of Sul7s are shown in the middle.

**Figure 6:**
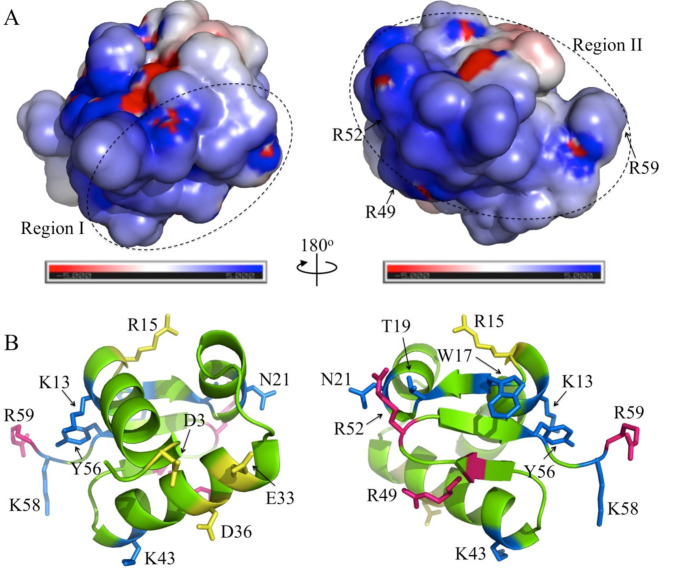
DNA binding surface of Sul7s. (**A**) The two opposite surfaces containing the residues of Sul7s that display large chemical shift perturbation (CSP), colored by electrostatic potential. Blue, positively charged; red, negatively charged; white, neutral. Region I and region II are indicated by ellipses in dashed line, respectively. Three key residues for ssDNA binding, i.e., R49, R52, and R59, are also indicated and labeled. (**B**) The representative conformer of the NMR structure of Sul7s is shown in ribbon in the same orientation as in (**A**). Residues included in the mutation analyses are shown in sticks and labeled. The residues that the mutations led to a severe, a moderate, and little decrease in DNA binding affinity of Sul7s, shown in pink, blue, and yellow, respectively.

The DNA-binding surface of Sul7s was determined by monitoring signal changes in ^1^H-^15^N HSQC spectra following titration of the protein with a 20-nt ssDNA (S20-F). During the titration, no significant backbone dynamic changes were found, as shown in the hetero-nuclear NOE experiments (Figure 5A). Changes were observed for the amide cross peaks of residues involved in DNA binding and the chemical shift perturbation (CSP) is plotted versus the residue number in Figure 5B. The residues with CSPs above the averaged CSP are considered to be located on the ssDNA binding surface, and those with CSPs larger than the averaged CSP plus the standard deviation are likely to contact directly to the ssDNA. More than one-third of the residues in Sul7s showed significant chemical shift changes in the HSQC spectra during ssDNA titration, indicating the presence of a large ssDNA-binding surface on the protein. As shown in Figure 6A, the residues involved in ssDNA-binding are mainly from two regions (regions I and II), located nearly on the two opposite sides on Sul7s. On one side, region I is composed of the perturbed residues from helix α3 (A31, E33, V34, D36, A37, and L38) and the N-terminus (D3), which is rich in acidic and hydrophobic residues (Figure 6A,B). On the other side, the much larger region II contains perturbed residues located in the β-sheet (β1: W17 and T19; β2: G48 and R49; W1: R52; β3: F54, Y55 and Y56; and W2: K58 and R59) and in the bundle of the helices (α1: K13 and R15; α2: F20 and N21; and α3: K43) (Figure 6A,B). Indeed, this region constitutes a large positive charged surface of Sul7s, owing to the abundance of lysines and arginines, and is therefore supposed to contribute predominantly to the ssDNA binding.

Mutation analyses of the residues with significant chemical shift perturbations were then performed to investigate their contribution to ssDNA binding. Fourteen mutants of Sul7s, i.e., D3A, K13A, R15A, W17A, T19G, N21A, E33A, D36A, K43A, R49A, R52A, Y56A, K58A, and R59A, were successfully overexpressed in *E. coli* and purified (Figure S5). The mutation sites cover all of the secondary structures of Sul7s. The binding affinities of the mutants to ssDNA S30 were then determined by BLI assays (Table 2 and Figure S6). As compared to wild-type Sul7s, all of the mutant proteins, except for R15A and E33A, showed a decrease in binding affinity for S30, confirming the large DNA interface of Sul7s. R15A showed a largely unchanged binding affinity, while E33A showed a moderately increased binding affinity to S30 as compared to the wild-type Sul7s. D3 and D36, the two acidic residues in region I, appeared to contribute slightly to ssDNA binding by the protein since mutation of the two residues led to a marginal decrease in the DNA binding affinity. Therefore, region I may not be directly involved in DNA binding by Sul7s, and the chemical perturbations of the acidic residues may have resulted from the rearrangement of the side chains on the protein surface upon ssDNA binding. In remarkable contrast to region I, mutations of the residues in region II displayed a great impact on the binding of Sul7s to ssDNA. The mutation of K13, W17, T19, N21A, K43, or K58 reduced the affinity of the protein to S30 by ~6.2, 2.2, 2.9, 1.8, 5.7, or 5.5 fold, respectively. Intriguingly, the decrease in the ssDNA binding affinity of these mutants was mainly due to a decrease in the association rate, and not a change in dissociation rate, suggesting that they serve important roles in ssDNA recognition. Notably, the binding by R49A, R52A, and R59A to S30 was barely detected even at protein concentrations up to 80 μM (Figure S6). Therefore, we conclude that the large ssDNA binding surface exists on one side of Sul7s (region II), which consists of residues from helices α1, α2, and α3 and strands β1, β2, and β3 as well as the wings W1 and W2, with the two wings and strand β2 serving crucial roles.

The structure-based sequence alignment of Sul7s and its structural homologues shows no conservation in DNA contacting residues or their distribution pattern (Figure 4E). For Sul7s, the β2-strand and the wings W1 and W2 are crucially important while α3-helix only serves a minor role in DNA binding (Figure 4E and Figure 6B). By contrast, the corresponding α3-helix in other proteins makes multiple contacts with bound DNA, and was regarded as the recognition helix in a number of wHTH proteins [30]. The key roles of the β2-strand and wing W1 in DNA binding were reported for some wHTH proteins, e.g., the 76-residue DNA-binding domain of human RFX1 (hRFX1; PDB code: 1DP7) [39], which is not identified as the structural homolog of Sul7s by DALI. The strands β2 and β3 and wing W1 of hRFX1 make extensive contacts with the major groove of one half-site of the symmetric X-box, while a single side chain from α3-helix interacts with the minor groove of the other half-site, as found in DNA binding by Sul7s. However, it should be noted that the essentiality of wing W2 in DNA binding, as observed for Sul7s, is unprecedented, presumably underlining the structural basis for the preference of different wHTH proteins for either ss- or dsDNA. Although K58 and R59 of W2 are not conserved in the Sul7s family members, lysine and arginine residues are found at the corresponding positions in other wHTH proteins possibly interacting with ssDNA, e.g., SsoPBP2 and SsoCDC6-3, further supporting the potential importance of W2 in ssDNA binding.

## 3. Discussion

The winged helix superfamily contains a large number of structurally related nucleic acid-binding protein families. The biological functions of the members of this superfamily are extremely diverse, spanning from sequence recognition in transcription factors, bulldozer-like strand separation in helicases to mediating protein–protein interaction [40]. While the winged helix proteins are often shown to bind dsDNA, few have been reported to bind ssDNA, e.g., LP1413 from *Staphylococcus aureus*, a winged helix-turn-helix single-stranded DNA binding protein [41]. In the present work, we report the identification and characterization of Sul7s, a novel single-stranded DNA binding protein family of winged helix protein, highly conserved in hyperthermophilic archaea of the *Sulfolobaceae*.

Solution structure shows that Sul7s from *S. islandicus* folds into a globular structure composed of a right-handed three-helix bundle and a three-strand antiparallel β-sheet, a signature topology of winged helix proteins (Figure 4A). The affinity of Sul7s for ssDNA is 15-fold greater than that for dsDNA in vitro (Figure 2). In addition, Sul7s uses a novel strategy in ssDNA recognition, which involves a large interface (region II) consisting of several residues from the β-sheet and the bundle of the α-helices close to the C-terminus (Figure 6). Among these residues, three arginines (R49A, R52A and R59) serve crucial roles in DNA interaction, suggesting that the two short wings are essential for the preferential ssDNA binding. The wing-based strategy in DNA recognition has also been reported for human transcription regulatory factor (RFXI) [39]. However, only wing W1 in RFX1 was shown to make most of the contacts with DNA in the major groove in the co-crystal of RFX1-DNA complexes. On the other hand, the DNA binding pattern of Sul7s differs distinctively from the helix-based DNA recognition strategy employed by a large portion of winged helix proteins, represented by HNF-3γ, a liver-specific transcription factor functioning in cell differentiation and tissue-specific gene expression [30,40]. The crystal structure of the HNF-3/fork head DNA-recognition motif in complex of DNA (PDB code: 1VTN) shows that helix α3, known as the recognition helix of the HTH motif, is located in the major groove of the DNA duplex and thus plays a key role in DNA binding [31]. Together, it is speculated that different DNA recognition strategies employed by various winged helix proteins may have been evolved in adaptation to the nature or conformation of their target DNA. Further, notably, K58 and R59 of wing W2 are not conserved among Sul7s homologs, suggesting the possible divergence in DNA binding modes and physiological functions of the members in this family.

Unlike SSB, Sul7s was shown to be non-essential in *S. islandicus* M.16.4 as revealed by the Tn5 transposon mutagenesis analysis [24], and no Sul7s homologues were encoded by the *S. acidocaldarius* strain, which grew robustly lacking SSB [42]. These results suggest that Sul7s is probably not required to complement the function of the canonical SSB. Our in vitro assays showed that Sul7s preferentially bound to ssDNA, preventing DNA annealing without effects on the stability and conformation of dsDNA. From these results, we infer that it may bind to and stabilize the transient single-stranded regions on chromosomal DNA, such as those generated by helicases in DNA replication, repair, or transcription, to facilitate processes requiring DNA to be single-stranded. The preference of Sul7s for T-rich sequences also points to potential asymmetry in DNA binding by Sul7s in vivo, which may lead to the unbound complementary A-rich strands more accessible to molecular machines. Together, Sul7s may serve different roles from canonical SSB in DNA replication or repair among species in *Sulfolobaceae*.

Similar sequence preference has also been reported for the other type of non-canonical ssDNA binding protein from hyperthermophilic crenarchaea, ThermoDBP-related protein2 (ThermoDBP-RP2) from *Aeropyrum pernix* [23]. ITC assays using the 21-mer DNA oligos showed that ThermoDBP-RP2 binds preferentially to a random ssDNA sequence or to homo-pyrimidine ssDNAs (dC_21_ and dT_21_), but very weakly or not at all to homo-purine ssDNAs (dA_21_ and dG_21_) in vitro [23]. Crystal structure of the ssDNA complex of ThermoDBP-RP2 revealed the potential clashes between a purine and the amino acid residue(s) at specific positions on the DNA interface of ThermoDBP-RP2, which was rich in lysines and arginines like Sul7s [23]. Alternatively or additionally, the tendency of homo-purine sequences to form higher-order structures, such as dG-quadruplexes [43] or poly-dA parallel helices [44], would also prohibit their interactions with ThermoDBP-RP2 [23]. These deductions could also help to explain why Sul7s preferred to bind dT_30_, but failed to bind dA_30_. By contrast, the canonical single-stranded DNA binding proteins, e.g., *E. coli* SSB and human RPA [2,3], show little apparent sequence preference. Therefore, the molecular basis of the sequence preference of Sul7s should be further explored in the future, which would greatly help to understand the distinctions in physiological functions between the canonical and non-canonical SSBs in Archaea.

## 4. Materials and Methods

### 4.1. Protein Overproduction and Purification

The gene encoding Sul7s from *S. islandicus* Rey15A (SiRe_1983) was commercially synthesized after codon optimization (see Supplementary Table S1 for the nucleotide sequence) and cloned into the NdeI/XhoI sites of pET30a, yielding plasmid pET30a-Sul7s. *E. coli* BL21 (DE3) cells harboring pSul7s were grown in Luria-Bertani broth containing 50 μg/mL kanamycin at 37 °C to an OD_600_ of ~0.6. Overproduction of Sul7s was induced by the addition of 0.8 mM isopropyl β-D-thiogalactopyranoside (IPTG), and incubation of the culture was continued for 3 h at 37 °C. The cells were harvested by centrifugation and resuspended in ice-cold buffer A (50 mM Tris-Cl, pH6.8, 100 mM NaCl and 5% (*w*/*v*) glycerol). After sonication at 4 °C, the lysate was centrifuged to remove cell debris. The cell extract was treated at 75 °C for 30 min and centrifuged. The supernatant was filtered through a 0.45-μm filter (Merck Millipore, Burlington, MA, USA) and loaded onto a 5-mL SP Sepharose column (GE Healthcare, Chicago, IL, USA) in an AKTA^TM^ chromatography system. The elution was performed with a linear salt gradient from 0 to 1 M NaCl in 50 mM Tris-Cl, pH6.8, and 5% (*w*/*v*) glycerol. Protein-containing fractions were pooled, concentrated, and loaded on to a Superdex 75 10/30 column (GE Healthcare) and proteins were eluted in buffer A. The protein concentration was determined by the Lowry method using bovine serum albumin as the standard.

### 4.2. Preparation of Mutant Proteins

Point mutations were introduced into Sul7s by site-directed mutagenesis using the Fast Mutagenesis System (TransGen Biotech, Beijing, China) with pET30a-Sul7s as the template (Supplementary Table S1). All the mutations were confirmed by DNA sequencing. The mutant constructs were transformed into *E. coli* BL21 (DE3) cells, and recombinant proteins were overproduced and purified as described for the wild-type protein.

### 4.3. Oligonucleotides

All oligonucleotides used in this work were synthesized commercially and purified by HPLC (Sangon, Shanghai, China), and the sequences were listed in Supplementary Table S1.

S30-F: 5′-biotin-TTTCTACCCTTTGGTGCTAATGCCCATACT
S30-R: 5′-AGTATGGGCATTAGCACCAAAGGGTAGAAA
A30: 5′-biotin-AAAAAAAAAAAAAAAAAAAAAAAAAAAAAA
T30: 5′-biotin-TTTTTTTTTTTTTTTTTTTTTTTTTTTTTTTTT
C30: 5′-biotin-AAAAAAAAAAAAAAAAAAAAAAAAAAAAAA
G-rich30: 5′-biotin-CTGGGGGCTGGGGGCTGGGGGCTGGGGGCT
AT26: 5′-ATATATATATATATATATATATATAT
S32-F: 5′-6-FAM-AGGGTTCTTTGTGGCGGCGTCATCTGTGCTTC
S32-R: 5′-GAAGCACAGATGACGCCGCCACAAAGAACCCT-BHQ1
S20-F: 5′-GTAGTCAGACACAGTAGTTC

### 4.4. Analytical Ultracentrifugation

Sedimentation velocity was determined in a Beckman Coulter ProteomeLab XL-I analytical ultracentrifuge using an An-50Ti rotor. The sedimentation of samples was monitored by absorbance at 280 nm. Solution density and viscosity were calculated using SEDNTERP software. Samples (400 μL) were centrifuged in 12 mm standard double sector cells at 20 °C and 50,000 rpm. All experiments were carried out in 50 mM Tris–HCl, pH 6.8, 50 mM NaCl.

### 4.5. Circular Dichroism (CD)

Far-UV CD spectra (260–190 nm) were recorded by using a Jasco J-715 spectropolarimeter equipped with a PTC-423S/15 Peltier temperature controller. CD measurements were carried out using a 0.1-cm-pathlength cell and a protein concentration of 0.1 mg/mL in a 10 mM potassium phosphate buffer, pH 7.4, at indicated temperatures in Figure 1D.

### 4.6. Biolayer Interferometry (BLI) Assays

BLI experiments were performed at 30 °C with a speed of 1000 rpm on the Octet Red96 system (ForteBio). To determine the binding of Sul7s to ssDNA and dsDNA, biotin-labeled DNAs (~0.3 nm; Supplementary Table S1) were immobilized onto streptavidin biosensors. Sul7s proteins were diluted in running buffer (50 mM Tris-Cl, pH7.5, 100 mM NaCl, 1 mM EDTA, and 0.02% (*v*/*v*) Tween-20) to indicated concentrations. The biosensors were equilibrated in the running buffer for 60 s (baseline), and then incubated for 120 s with Sul7s at various concentrations in parallel, followed by dissociation for 120 s in the running buffer. The biosensors were washed with 1 M NaCl for 10 s and the running buffer for 10 s. This process was repeated twice to remove the bound Sul7s and regenerate the DNA biosensors. The rate constants of association (*k_a_*) and dissociation (*k_d_*), and the equilibrium dissociation constant (*K_D_*) were derived using a 1:1 binding model (Fortebio Data Analysis 8.5 Software). The binding kinetics for all the oligonucleotides was determined in triplicate.

### 4.7. Strand-Annealing Assay

S32-F and S32-R were mixed at 0.1 μM each in the presence or absence of 20 μM Sul7s in 50 mM Tris–HCl, pH 6.8, and 50 mM NaCl. The mixtures were incubated at 30 °C and the fluorescence was measured at intervals using a fluorescence spectrophotometer. Changes in normalized fluorescence intensity for each sample were calculated and plotted against time.

### 4.8. Thermal Denaturation of dsDNA

Sul7s was incubated at 25 °C for 10 min with a 26-bp dsDNA fragment, prepared by annealing AT26 (Table S1), in 20 mM potassium phosphate, pH 7.4, and 100 mM NaCl. Thermal denaturation of the DNA was monitored at A260 in a temperature range from 25 to 95 °C on a DU800 UV/Visible spectrophotometer (Beckman, Irvine, CA, USA). The melting temperature (Tm) was obtained using DU800 software.

### 4.9. Nick Closure Assays

The assays were performed as described previously [45]. Briefly, plasmid pBR322 containing a single nick (0.3 μg) was incubated with recombinant Sul7s at protein/DNA mass ratios of 0, 1, 2, 4, 8, 16, 32, 64, 128, and 256, respectively, for 10 min at 25 °C in a final volume of 15 μL. After ligation by T4 DNA ligase (Fermentas, Waltham, MA, USA) for 2 min at 25 °C, samples were deproteinized and analyzed by agarose gel electrophoresis. Gels were stained in 0.5 μg/mL ethidium bromide and imaged under UV light.

### 4.10. NMR Spectroscopy

Sul7s was uniformly isotope-labeled with ^15^N/^13^C by growing the recombinant strain in M9 minimal medium containing ^15^NH_4_Cl and ^13^C6-glucose (Cambridge Isotope Laboratories Inc., Tewksbury, MA, USA) as the sole nitrogen and carbon source, respectively. The labeled protein was purified as described above. Aliquots of Sul7s (~0.7 mM) in 20 mM potassium phosphate buffer (pH 7.0) containing 30 mM NaCl and 0.02% (*w*/*v*) NaN_3_ were subjected to NMR analysis. All NMR spectra were acquired at 298 K on an Agilent DD2 600 spectrometer equipped with a cryo-probe. The sequential backbone resonance assignments were achieved by using standard triple-resonance experiments: HNCACB, CBCA(CO)NH, and HNCO [46,47]. Non-exchangeable side chain resonance assignments were carried out with the help of the following spectra: HBHA(CO)NH, HCCH-TOCSY, and ^13^C-edited NOESY [47,48]. Inter-proton distance restraints were derived from 3D NOESY spectra (all with 100 ms mixing time): ^13^C-edited NOESY and ^15^N-edited NOESY. The spectra were processed with the program of NMRPipe [49] and analyzed with CcpNmr [28]. Structures calculations and NOE assignment were performed simultaneously by using the program CNS [50,51] and ARIA2 [27]. Backbone dihedral angle restraints (φ and ψ angles) were derived using the program DANGLE [52] incorporated in the CcpNmr software package. A total of 100 structures were calculated and the 20 structures with the lowest total energy were selected to perform a refinement procedure in water. The refined structure ensemble was selected to represent the solution structure of Sul7s. The atomic coordinates of Sul7s have been deposited in the Protein Data Bank with an accession code of 7BZH and the chemical shift assignments of the protein has been deposited in the Biological Magnetic Resonance Data Bank with an accession number of 36,352 (http://www.bmrb.wisc.edu accessed on 14 February 2022). The protein structure ensemble was displayed and analyzed with PyMol (http://www.pymol.org/ accessed on 14 February 2022).

Binding of Sul7s to a 20-nt DNA oligonucleotide S20-F in 20 mM potassium phosphate buffer (pH 7.0) containing 30 mM NaCl and 0.02% (*w*/*v*) NaN_3_ was monitored with ^1^H-^15^N HSQC using ^15^N/^13^C-labeled Sul7s (0.7 mM) (Figure S7). The backbone assignments of the Sul7s-ssDNA complex were obtained in CBCA(CO)NH and HNCA experiments, in which ^15^N/^13^C-labeled Sul7s (0.7mM) was mixed with a slight excess amount of the ssDNA. The ^15^N-^1^H hetero-nuclear NOE experiments were performed with and without proton saturation during the relaxation delay (5 s) and the saturation time is 3 s to build the NOE. ^15^N-^1^H NOEs are reported as the ratio of *I_noe_*/*I_ref_*, where *I_noe_* and *I_ref_* are the peak intensities in the saturated experiment and non-saturated experiment, respectively. During titration with the ssDNA, the overall chemical shift change for each residue of Sul7s was determined by the following equation: $\mathrm{CSP}=\sqrt{({\mathrm{\Delta H}}^{2}+{\left(\mathrm{\Delta N}/5\right)}^{2}}$, in which CSP stands for the overall chemical shift perturbation, ΔH and ΔN are the chemical shift differences between the apo and ssDNA-bound states of Sul7s for proton and nitrogen, respectively. The standard deviation of the CSP was calculated according to the equation: $\mathrm{STD}\;=\sqrt{\frac{\sum {\left(CSP_{res}-CSP_{avg}\right)}^{2}}{N_{res}}}$, where CSP_res_ is the residue-specific CSP, CSP_avg_ is the averaged CSP of all residues, and N_res_ is the number of residues used in this calculation.

## Figures and Tables

**Table 1:** Structural statistics for the ensemble of the 20 lowest energy structures of Sul7s in aqueous solution.

**Total number of distance restraints** **^1^**	1388
intra residual (|i-j| = 0)	565
sequential (|i-j| = 1)	246
medium range (1<|i-j|<5)	134
long range (|i-j|> = 5)	264
ambiguous restraints	179
**Dihedral angle restraints** **^2^**	
Φ	52
Ψ	51
**Average number of restraints per residue**	25.3
**Mean rmsd from idealized covalent geometry** **^1^**	
bond (Å)	3.252 × 10^−3^ ± 8.766 × 10^−^^5^
angle (^o^)	4.781 × 10^−^^1^ ± 8.692 × 10^−3^
improper (^o^)	1.042 ± 6.632 × 10^−^^2^
**Mean rmsd from the experimental restraints** **^1^**	
dihedral angle (^o^)	3.400 × 10^−^^1^ ± 8.356 × 10^−^^2^
distance (Å)	2.078 × 10^−^^2^ ± 1.678 × 10^−3^
**Ramachandran plot** **^3^**	
% residues in the most favorable regions	93%
allowed regions	7%
disallowed regions	0%
**Atomic rmsd** **^1^**	
backbone	0.426 ± 0.118
heavy atoms	0.967 ± 0.142

^1^ Statistics were derived from software of Aria2.3 [27]. ^2^ Statistics were derived from CcpNmr software package [28]. ^3^ Statistics were derived from Molprobity [29].

**Table 2:** Kinetic parameters of the binding of wild type and mutant Sul7s proteins to S30 as determined by BLI ^1^.

Mutants	Binding Affinity *K*_D_ (M)	Association Rate *k*_a_ (M^−1^ s^−1^)	Dissociation Rate *k*_d_ (s^−1^)
D3A	6.01 ± 0.80 × 10^−6^	6.91 ± 0.65 × 10^2^	4.09 ± 0.16 × 10^−3^
K13A	3.04 ± 0.54 × 10^−5^	4.28 ± 0.27 × 10^2^	1.32 ± 0.31 × 10^−2^
R15A	4.67 ± 0.53 × 10^−6^	1.24 ± 0.40 × 10^2^	5.80 ± 0.85 × 10^−3^
W17A	1.07 ± 0.35 × 10^−5^	5.84 ± 0.74 × 10^2^	6.29 ± 1.00 × 10^−3^
T19G	1.41 ± 0.40 × 10^−5^	4.83 ± 0.20 × 10^2^	6.74 ± 1.62 × 10^−3^
N21A	8.92 ± 0.29 × 10^−6^	8.39 ± 0.37 × 10^2^	7.50 ± 0.57 × 10^−3^
E33A	1.21 ± 0.09 × 10^−6^	4.57 ± 0.59 × 10^3^	5.56 ± 1.11 × 10^−3^
D36A	8.83 ± 1.15 × 10^−5^	3.74 ± 0.12 × 10^2^	3.29 ± 0.33 × 10^−2^
K43A	2.79 ± 0.50 × 10^−5^	5.01 ± 0.22 × 10^2^	1.41 ± 0.31 × 10^−2^
R49A	ND ^2^	ND ^2^	ND ^2^
R52A	ND ^2^	ND ^2^	ND ^2^
Y56A	1.56 ± 0.67 × 10^−5^	4.94 ± 0.78 × 10^2^	7.17 ± 2.10 × 10^−3^
K58A	2.69 ± 0.42 × 10^−5^	3.54 ± 0.04 × 10^2^	9.52 ± 1.61 × 10^−3^
R59A	ND ^2^	ND ^2^	ND ^2^

^1^ Data shown here is an average of the data sets from two independent experiments. ^2^ ND, Not detected.

## Data Availability

Chemical shift assignments of *S.* *islandicus* Sul7s have been deposited in the BioMagResBank under accession number 36352. The atomic coordinates of *S.* *islandicus* Sul7s and all restraints have been deposited in the Protein Data Bank with accession code 7BZH.

## Supplementary Materials

The following supporting information can be downloaded at: https://www.mdpi.com/article/10.3390/ijms23073455/s1.

## References

[1] Mushegian A., Koonin E.V. (1996). A minimal gene set for cellular life derived by comparison of complete bacterial genomes. Proc. Natl. Acad. Sci. USA.

[2] Antony E., Lohman T.M. (2019). Dynamics of *E. coli* single stranded DNA binding (SSB) protein-DNA complexes. Semin. Cell Dev. Biol..

[3] Byrne B.M., Oakley G.G. (2019). Replication protein A, the laxative that keeps DNA regular: The importance of RPA phosphorylation in maintaining genome stability. Semin. Cell Dev. Biol..

[4] Sun S., Shamoo Y. (2003). Biochemical Characterization of Interactions between DNA Polymerase and Single-stranded DNA-binding Protein in Bacteriophage RB69. J. Biol. Chem..

[5] Shamoo Y., Friedman A.M., Parsons M.R., Konigsberg W.H., Steitz T.A. (1995). Crystal structure of a replication fork single-stranded DNA binding protein (T4 gp32) complexed to DNA. Nature.

[6] Hollis T., Stattel J.M., Walther D.S., Richardson C.C., Ellenberger T. (2001). Structure of the gene 2.5 protein, a single-stranded DNA binding protein encoded by bacteriophage T7. Proc. Natl. Acad. Sci. USA.

[7] Meyer R.R., Laine P.S. (1990). The single-stranded DNA-binding protein of Escherichia coli. Microbiol. Rev..

[8] Wold M.S. (1997). Replication Protein A: A Heterotrimeric, Single-Stranded DNA-Binding Protein Required for Eukaryotic DNA Metabolism. Annu. Rev. Biochem..

[9] Shereda R.D., Kozlov A.G., Lohman T.M., Cox M.M., Keck J.L. (2008). SSB as an Organizer/Mobilizer of Genome Maintenance Complexes. Crit. Rev. Biochem. Mol..

[10] Zou Y., Liu Y., Wu X., Shell S.M. (2006). Functions of human replication protein A (RPA): From DNA replication to DNA damage and stress responses. J. Cell. Physiol..

[11] Fanning E., Klimovich V., Nager A.R. (2006). A dynamic model for replication protein A (RPA) function in DNA processing pathways. Nucleic Acids Res..

[12] Suck D. (1997). Common fold, common function, common origin?. Nat. Struct. Biol..

[13] Theobald D.L., Mitton-Fry R.M., Wuttke D.S. (2003). Nucleic Acid Recognition by OB-Fold Proteins. Annu. Rev. Biophys. Biomol. Struct..

[14] Raghunathan S., Kozlov A.G., Lohman T.M., Waksman G. (2000). Structure of the DNA binding domain of E. coli SSB bound to ssDNA. Nat. Struct. Biol..

[15] Bochkarev A., Bochkareva E., Frappier L., Edwards A.M. (1999). The crystal structure of the complex of replication protein A subunits RPA32 and RPA14 reveals a mechanism for single-stranded DNA binding. EMBO J..

[16] Bochkarev A., Pfuetzner R.A., Edwards A.M., Frappier L. (1997). Structure of the single-stranded-DNA-binding domain of replication protein A bound to DNA. Nature.

[17] Komori K., Ishino Y. (2001). Replication Protein A in Pyrococcus furiosus Is Involved in Homologous DNA Recombination. J. Biol. Chem..

[18] White M. (2003). Archaeal DNA repair: Paradigms and puzzles. Biochem. Soc. Trans..

[19] Wadsworth R.I., White M.F. (2001). Identification and properties of the crenarchaeal single-stranded DNA binding protein from Sulfolobus solfataricus. Nucleic Acids Res..

[20] Touma C., Kariawasam R., Gimenez A.X., Bernardo R.E., Ashton N.W., Adams M.N., Paquet N., Croll T.I., O’Byrne K.J., Richard D.J. (2016). A structural analysis of DNA binding by hSSB1 (NABP2/OBFC2B) in solution. Nucleic Acids Res..

[21] Kerr I.D., Wadsworth R.I.M., Cubeddu L., Blankenfeldt W., Naismith J.H., White M.F. (2003). Insights into ssDNA recognition by the OB fold from a structural and thermodynamic study of Sulfolobus SSB protein. EMBO J..

[22] Paytubi S., McMahon S.A., Graham S., Liu H., Botting C.H., Makarova K.S., Koonin E.V., Naismith J.H., White M.F. (2012). Displacement of the canonical single-stranded DNA-binding protein in the Thermoproteales. Proc. Natl. Acad. Sci. USA.

[23] Ghalei H., Von Moeller H., Eppers D., Sohmen D., Wilson D., Loll B., Wahl M.C. (2014). Entrapment of DNA in an intersubunit tunnel system of a single-stranded DNA-binding protein. Nucleic Acids Res..

[24] Zhang C., Phillips A.P.R., Wipfler R.L., Olsen G.J., Whitaker R.J. (2018). The essential genome of the crenarchaeal model *Sulfolobus islandicus*. Nat. Commun..

[25] Suzuki S., Kurosawa N. (2019). Robust growth of archaeal cells lacking a canonical single-stranded DNA-binding protein. FEMS Microbiol. Lett..

[26] Van Dijk M., Mulders J., Poutsma A., Könst A.A.M., Lachmeijer A.A.M., Dekker A.G., Blankenstein M., Oudejans C.B.M. (2005). Maternal segregation of the Dutch preeclampsia locus at 10q22 with a new member of the winged helix gene family. Nat. Genet..

[27] Rieping W., Habeck M., Bardiaux B., Bernard A., Malliavin T.E., Nilges M. (2007). ARIA2: Automated NOE assignment and data integration in NMR structure calculation. Bioinformatics.

[28] Vranken W., Boucher W., Stevens T.J., Fogh R.H., Pajon A., Llinas M., Ulrich E.L., Markley J.L., Ionides J., Laue E.D. (2005). The CCPN data model for NMR spectroscopy: Development of a software pipeline. Proteins.

[29] Chen V.B., Arendall W.B., Headd J.J., Keedy D.A., Immormino R.M., Kapral G.J., Murray L.W., Richardson J.S., Richardson D.C. (2010). *MolProbity*: All-atom structure validation for macromolecular crystallography. Acta Crystallogr. D.

[30] Gajiwala K.S., Burley S.K. (2000). Winged helix proteins. Curr. Opin. Struct. Biol..

[31] Clark K.L., Halay E.D., Lai E., Burley S.K. (1993). Co-crystal structure of the HNF-3/fork head DNA-recognition motif resembles histone H5. Nature.

[32] Hasegawa H., Holm L. (2009). Advances and pitfalls of protein structural alignment. Curr. Opin. Struct. Biol..

[33] Light S.H., Cahoon L.A., Halavaty A.S., Freitag N.E., Anderson W.F. (2016). Structure to function of an alpha-glucan metabolic pathway that promotes Listeria monocytogenes pathogenesis. Nat. Microbiol..

[34] Shi J., Wen A., Zhao M., You L., Zhang Y., Feng Y. (2019). Structural basis of sigma appropriation. Nucleic Acids Res..

[35] Ha S.C., Lokanath N.K., Van Quyen D., Wu C.A., Lowenhaupt K., Rich A., Kim Y.-G., Kim K.K. (2004). A poxvirus protein forms a complex with left-handed Z-DNA: Crystal structure of a Yatapoxvirus Zalpha bound to DNA. Proc. Natl. Acad. Sci. USA.

[36] Yan J., Beattie T.R., Rojas A.L., Schermerhorn K., Gristwood T., Trinidad J.C., Albers S.V., Roversi P., Gardner A.F., Abrescia N.G.A. (2017). Identification and characterization of a heterotrimeric archaeal DNA polymerase holoenzyme. Nat. Commun..

[37] Ali T., Coles P., Stevens T.J., Stott K., Thomas J.O. (2004). Two Homologous Domains of Similar Structure but Different Stability in the Yeast Linker Histone, Hho1p. J. Mol. Biol..

[38] Dueber E.L.C., Corn J.E., Bell S.D., Berger J.M. (2007). Replication Origin Recognition and Deformation by a Heterodimeric Archaeal Orc1 Complex. Science.

[39] Gajiwala K.S., Chen H., Cornille F., Roques B.P., Reith W., Mach B., Burley S. (2000). Structure of the winged-helix protein hRFX1 reveals a new mode of DNA binding. Nature.

[40] Harami G.M., Gyimesi M., Kovács M. (2013). From keys to bulldozers: Expanding roles for winged helix domains in nucleic-acid-binding proteins. Trends Biochem. Sci..

[41] Sanchis I.M., Pigli Y.Z., Rice P.A. (2018). Crystal Structure of an Unusual Single-Stranded DNA-Binding Protein Encoded by Staphylococcal Cassette Chromosome Elements. Structure.

[42] Suzuki S., Kurosawa N. (2017). Development of the Multiple Gene Knockout System with One-Step PCR in Thermoacidophilic Crenarchaeon *Sulfolobus acidocaldarius*. Archaea.

[43] Gellert M., Lipsett M.N., Davies D.R. (1962). Helix formation by guanylic acid. Proc. Natl. Acad. Sci. USA.

[44] Saenger W., Riecke J., Suck D. (1975). A structural model for the polyadenylic acid single helix. J. Mol. Biol..

[45] Zhang Z., Zhao M., Wang L., Chen Y., Dong Y., Gong Y., Huang L. (2017). Roles of Leu28 side chain intercalation in the interaction between Cren7 and DNA. Biochem. J..

[46] Ikura M., Kay L.E., Bax A. (1990). A Novel Approach for Sequential Assignment of 1H, 13C, and 15N Spectra of Proteins: Heteronuclear Triple-Resonance Three-Dimensional NMR Spectroscopy. Application to Calmodulin. Biochemistry.

[47] Bax A., Ikura M., Kay L.E., Barbato G., Spera S. (1991). Multidimensional Triple Resonance NMR Spectroscopy of Isotopically Uniformly Enriched Proteins: A Powerful New Strategy for Structure Determination. Ciba Found Symp..

[48] Bax A., Ikura M. (1991). An efficient 3D NMR technique for correlating the proton and15N backbone amide resonances with the α-carbon of the preceding residue in uniformly15N/13C enriched proteins. J. Biomol. NMR.

[49] Delaglio F., Grzesiek S., Vuister G.W., Zhu G., Pfeifer J., Bax A. (1995). NMRPipe: A multidimensional spectral processing system based on UNIX pipes. J. Biomol. NMR.

[50] Brunger A.T., Adams P., Clore G.M., Delano W.L., Gros P., Grosse-Kunstleve R.W., Jiang J.S., Kuszewski J., Nilges M., Pannu N.S. (1998). Crystallography & NMR System: A New Software Suite for Macromolecular Structure Determination. Acta Crystallogr. Sect. D Biol. Crystallogr..

[51] Brunger A.T. (2007). Version 1.2 of the Crystallography and NMR system. Nat. Protoc..

[52] Cheung M.-S., Maguire M.L., Stevens T.J., Broadhurst R.W. (2010). DANGLE: A Bayesian inferential method for predicting protein backbone dihedral angles and secondary structure. J. Magn. Reson..

