# Supplementary material for: A Novel Family of Winged-Helix Single-Stranded DNA-Binding Proteins from Archaea
Source: Int J Mol Sci. 2022 Mar 22;23(7):3455. doi: 10.3390/ijms23073455 (PMC8998557; doi:10.3390/ijms23073455)
Supplement: Supplementary file 1 [file ijms-23-03455-s001.zip › ijms-1618523-supplementary.pdf]

International Journal of *Molecular Sciences*

MDPI

Supplementary materials

Table S1. Nucleotide sequences of the commercially synthesized gene and primers.

Name	Sequence (5′-3′)
ORF of SiRe_1983	ATGGAAGATGTTAAACAGAGCGTTGAAAAAATTATTAAAGATCGT<br>GAATGGGTTACCTTTAATGATCTGCTGAAATATATTCCGTATCCGG<br>CACCGGAAGTTTATGATGCCCTGAGCCAGCTGATTAAAGAAAATA<br>AAGTTGGTCGTCGTGGTCGTTATTTTTATTATATCAAACGCTAA
D3A-F	GAGATATACATATGGAAGCAGTTAAACAGAGCG
D3A-R	TGCTTCCATATGTATATCTCCTTCTTAAAGTT
K13A-F	AGCGTTGAAAAAATTATTGCAGATCGTGAATGG
K13A-R	TGCAATAATTTTTTCAACGCTCTGTTTAACATC
R15A-F	GAAAAAATTATTAAAGATGCTGAATGGGTTACC
R15A-R	AGCATCTTTAATAATTTTTTCAACGCTCTGTTT
W17A-F	ATTATTAAAGATCGTGAAGCGGTTACCTTTAATG
W17A-R	CGCTTCACGATCTTTAATAATTTTTTCAACGC
T19G-F	AAAGATCGTGAATGGGTTGGCTTTAATGATCTG
T19G-R	GCCAACCCATTCACGATCTTTAATAATTTTTTC
N21A-F	CGTGAATGGGTTACCTTTGCAGATCTGCTGAAA
N21A-R	TGCAAAGGTAACCCATTCACGATCTTTAATAAT
E33A-F	ATTCCGTATCCGGCACCGGCAGTTTATGATGCC
E33A-R	TGCCGGTGCCGGATACGGAATATATTTCAGCAG
D36A-F	TTTAATGATCTGCTGAAAGCTATTCCGTATCCG
D36A-R	AGCTTTCAGCAGATCATTAAAGGTAACCCATTC
K43A-F	GCCCTGAGCCAGCTGATTGCAGAAAATAAAGTT
K43A-R	TGCAATCAGCTGGCTCAGGGCATCATAAACTTC
R49A-F	AAAGAAAATAAAGTTGGTGCTCGTGGTCGTTAT
R49A -R	AGCACCAACTTTATTTTCTTTAATCAGCTGGCT
R52A-F	AAAGTTGGTCGTCGTGGTGCTTATTTTTATTAT
R52A-R	AGCACCACGACGACCAACTTTATTTTCTTTAATC
Y56A-F	CGTGGTCGTTATTTTTATGCTATCAAACGCTAA
Y56A-R	AGCATAAAAATAACGACCACGACGACCAACTTT
K58A-F	CGTTATTTTTATTATATCGCACGCTAACTCGAG
K58A-R	TGCGATATAATAAAAATAACGACCACGACGACC
R59A-F	TATTTTTATTATATCAAAGCCTAACTCGAGCAC
R59A-R	GGCTTTGATATAATAAAAATAACGACCACGACG

Table S2. Primers used in construction of Sul7s deletion mutant strain of *S. islandicus*.

Name[a]	Sequence[b]
Sp-fwd	AAAGCTCCAGAAGTTTACGATGCATTATCCCAACTTATTAAGGA
Sp-rev	TAGCTCCTTAATAAGTTGGGATAATGCATCGTAAACTTCTGGAG
L-fwd	ACGCGTCGACATTACTCTCAACTACCTCAGAGAA
L-rev	CGAAAATCTAATAAAAGTTAGTTCTTGCAGTTTGTGTTAAAAAGGTCCGTATA
R-fwd	TATACGGACCTTTTTAACACAAACTGCAAGAACTAACTTTTATTAGATTTTCG
R-rev	ATAAGAATGCGGCCGCACGTAGTTGGTATAATTGCATTT\
Int-fwd	ATGGAAGATGTTAAACAGTCTGTAGAA
Int -rev	AGGTTGTAAAGAATCTCTTTGACAAT
Fl -fwd	AATAGCACAATTCGCTTCTGAG
Fl-rev	TGAGCTCTCAACTTAGCAAGAAT

[a] Sp, Spacer; L, Left-arm; R, Right-arm; fwd, forward primers; rev, reverse primers; Fl, Flanking primers; Int, Internal primers; [b] Restriction sites are underlined. Lowercase letters indicate bases to

be introduced to replace those in a wild-type gene.

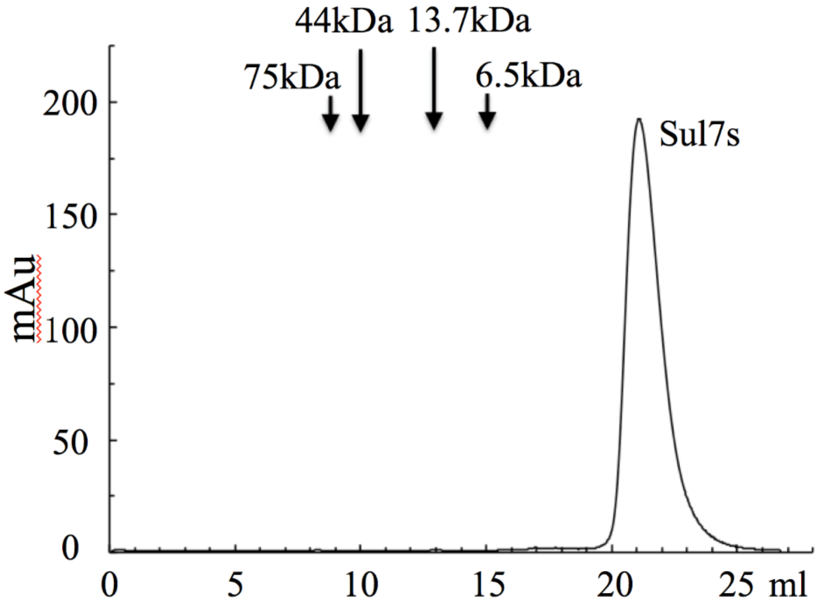

Figure S1. Gel-filtration chromatography of the recombinant Sul7s protein (3 mg protein in 1 mL volume). A 100 μL sample (5 mg/mL) of Sul7s was subjected to size exclusion chromatography in buffer A on a Superdex 75 column (GE healthcare) at a flow rate of 0.5 mL/min at 4 °C. Elution of the protein was monitored by absorbance at 280 nm. The void volume (v_o) and total volume (v_t) of the column were determined with blue dextran and xylene cyanol, respectively. The fractional retention (K_{av}) was calculated using the formula $K_{av} = (v_e–v_o)/(v_t–v_o)$, where v_e is the peak elution volume. A standard curve of K_{av} against log Mr was generated by using low molecular weight protein standards (GE healthcare).

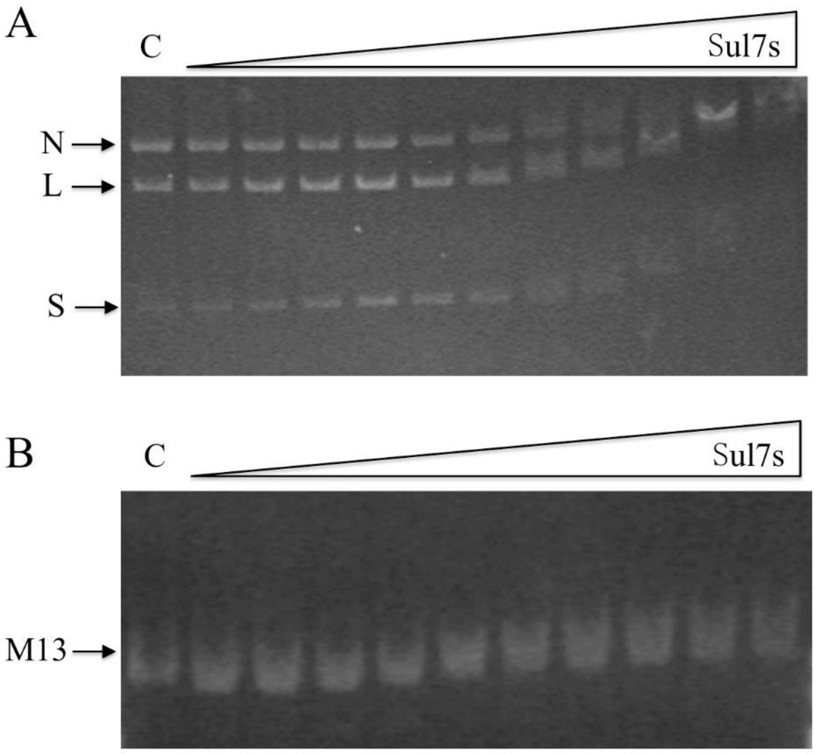

Figure S2. Agarose gel electrophoresis of plasmid pBR322 DNA (**A**) and M13 phage DNA (**B**) in the absence and presence of Sul7s. Sul7s was mixed with 300 ng DNA to yield Sul7s/DNA mass ratios of 1, 2, 4, 8, 16, 32, 64,128, 256 and 512 in 50 mM Tris–HCl, pH 6.8 and 50 mM NaCl. The mixtures

were incubated for 20 min at room temperature and then loaded onto the 1% agarose gel. Electrophoresis was at 80 V for 120 min at room temperature, depending on the desired separation. The gel was stained with ethidium bromide (0.5 μg/mL) and imaged under UV light. S, supercoiled DNA; L, linear DNA; N, singly nicked circular DNA; C, control DNA.

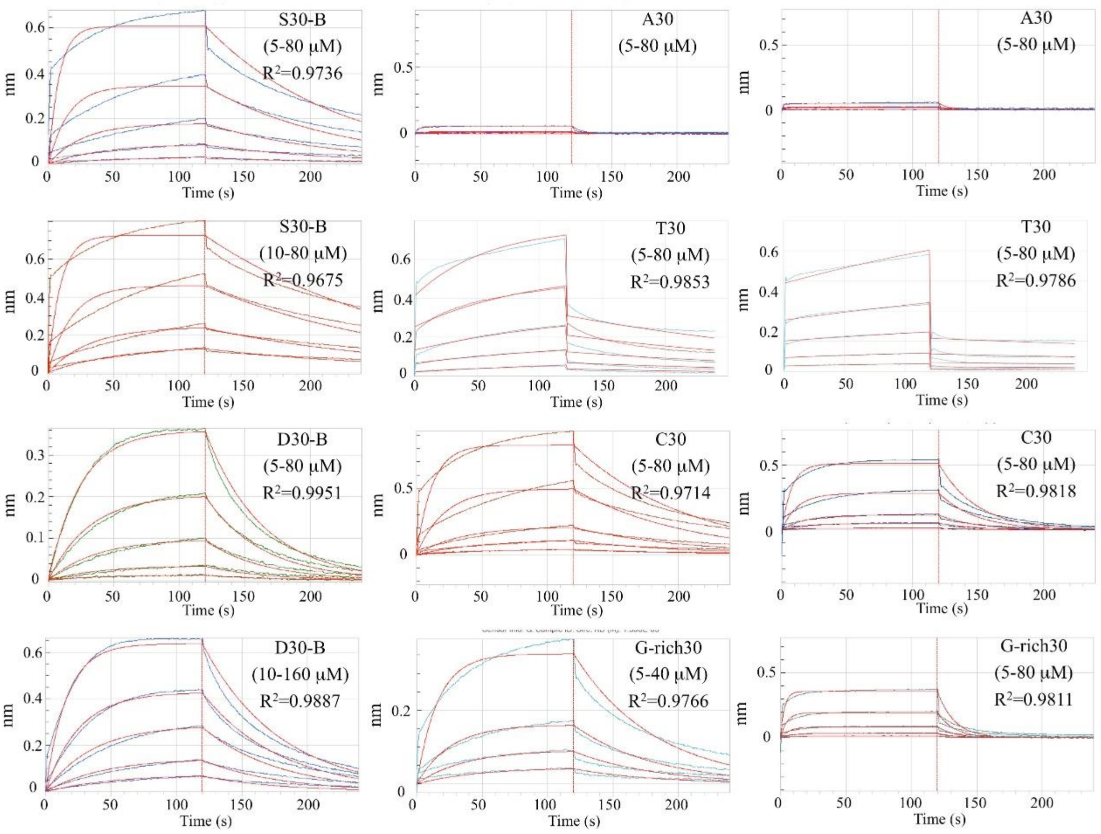

Figure S3. BLI assays of Sul7s binding to the 30-nt oligonucleotides (S30, A30, T30, C30 and G-rich30) and the 30-bp DNA duplex D30 with the same sequence to S30. The R^2 value for each fit is indicated.

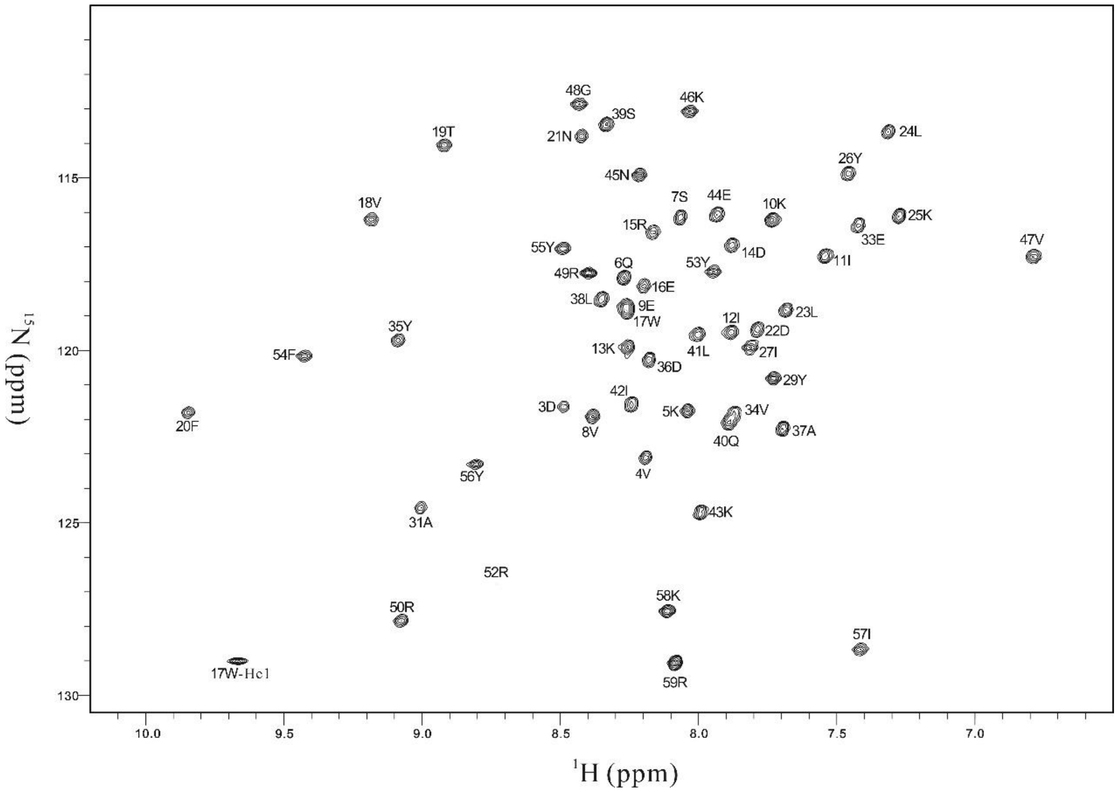

Figure S4. ^{1}H-^{15}N HSQC spectrum of Sul7s. All backbone amides showed well dispersed signals in this spectrum. Resonance assignments were labeled beside the signals.

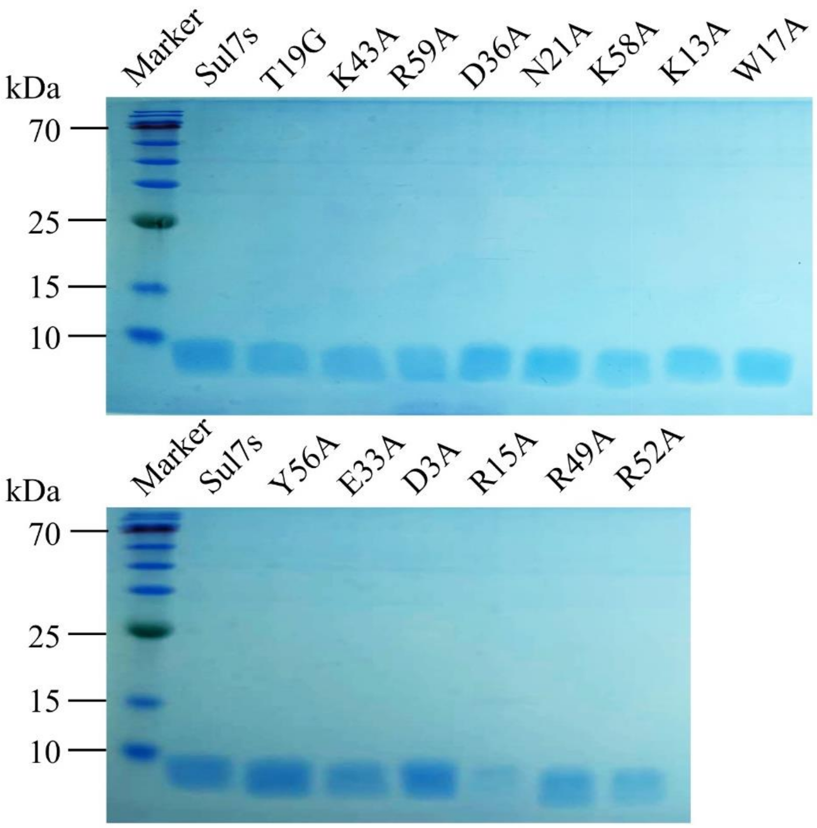

Figure S5. SDS-PAGE analyses of the mutant Sul7s proteins.

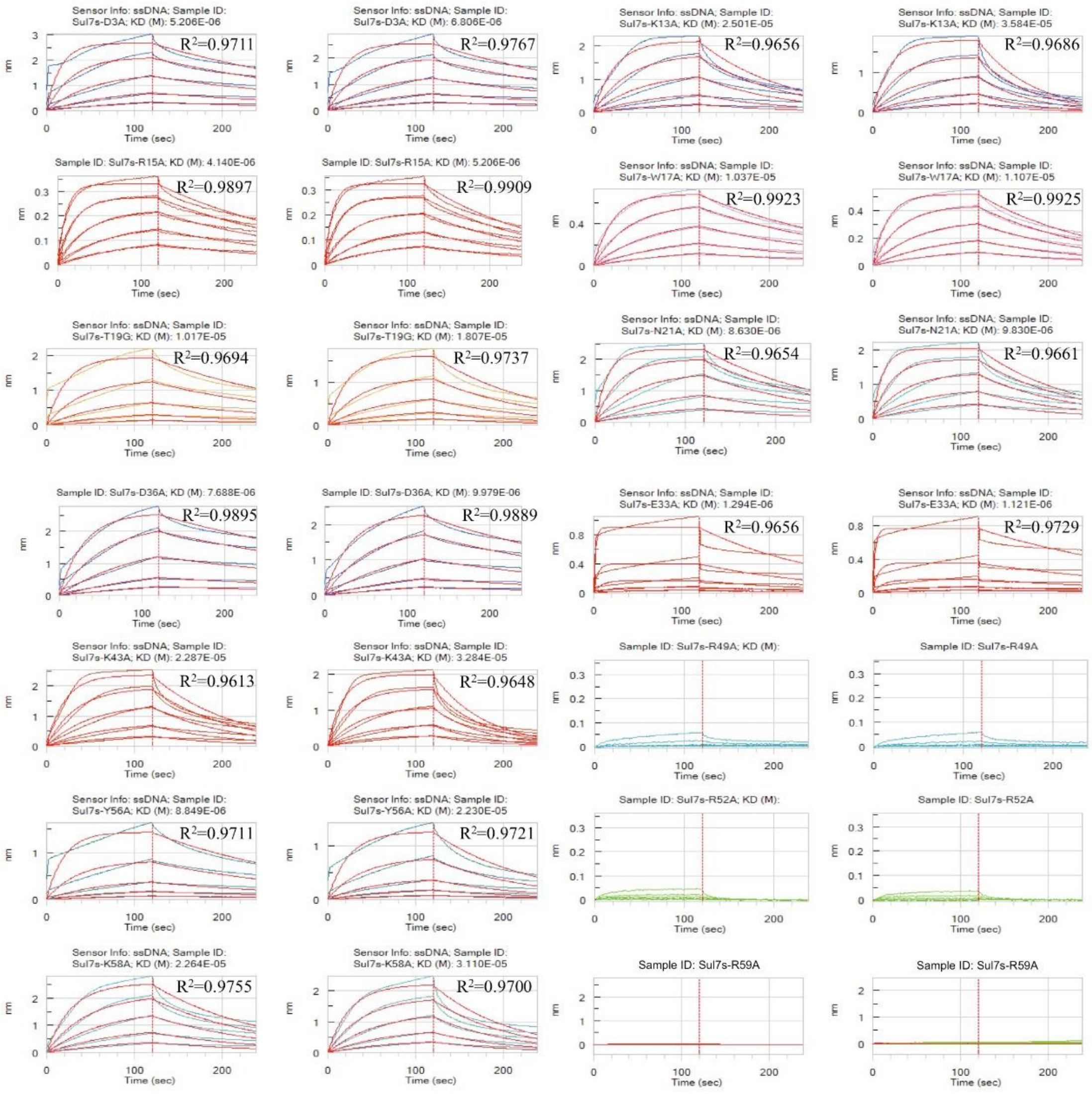

Figure S6. BLI assays of Sul7s mutants binding to the 30-nt oligonucleotides (S30). BLI data for different protein concentrations are shown, and the 1:1 model fit curves are in red. The range of the protein concentration for each mutant is 5-80 μM. The R^2 value for each fit is indicated.

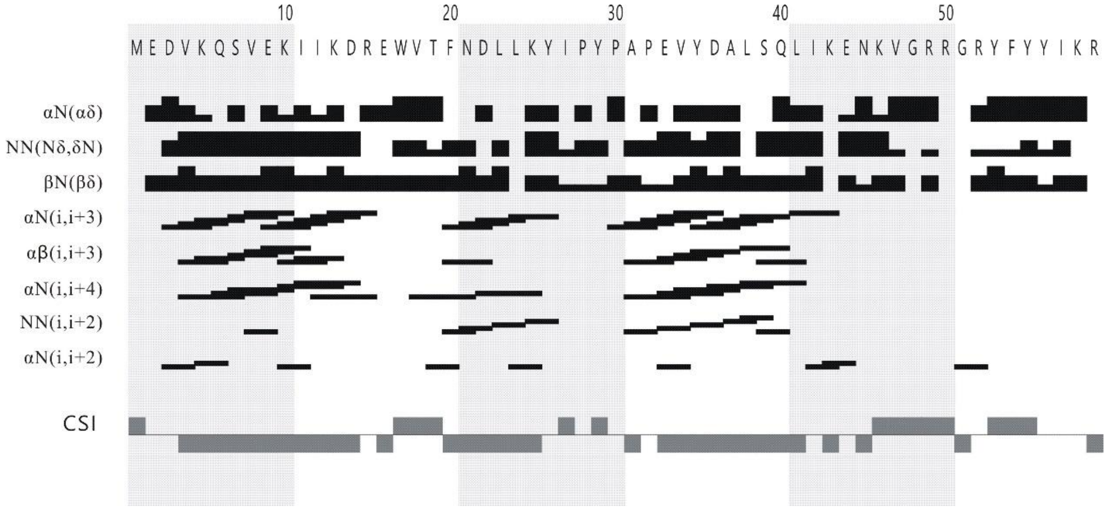

Figure S7. NOE pattern and CSI (Chemical Shift Index) of Sul7s derived from CcpNMR. Residue number was indicated on the top while the CSI were shown at the bottom as histogram of values of either 1 or -1. The two NOE connected atoms names and their residues numbers were listed on the

left while the connection was indicated as line between them with the thickness of the line as an indicator of the NOE intensity.
